# Oral Versus Intragastric Inoculation: Similar Pathways of *Trypanosoma cruzi* Experimental Infection? From Target Tissues, Parasite Evasion, and Immune Response

**DOI:** 10.3389/fimmu.2018.01734

**Published:** 2018-07-27

**Authors:** Juliana Barreto de Albuquerque, Danielle Silva dos Santos, Jens V. Stein, Juliana de Meis

**Affiliations:** ^1^Theodor Kocher Institute, University of Bern, Bern, Switzerland; ^2^Laboratory on Thymus Research, Oswaldo Cruz Institute, Oswaldo Cruz Foundation, Rio de Janeiro, Brazil; ^3^National Institute of Science and Technology on Neuroimmunomodulation (INCT-NIM), Rio de Janeiro, Brazil

**Keywords:** *Trypanosoma cruzi*, oral cavity, intragastric infection, immune response, T cell activation

## Abstract

Currently, oral infection is the most frequent transmission mechanism of Chagas disease in Brazil and others Latin American countries. This transmission pathway presents increased mortality rate in the first 2 weeks, which is higher than the calculated mortality after the biting of infected insect vectors. Thus, the oral route of *Trypanosoma cruzi* infection, and the consequences in the host must be taken into account when thinking on the mechanisms underlying the natural history of the disease. Distinct routes of parasite entry may differentially affect immune circuits, stimulating regional immune responses that impact on the overall profile of the host protective immunity. Experimental studies related to oral infection usually comprise inoculation in the mouth (oral infection, OI) or gavage (gastrointestinal infection, GI), being often considered as similar routes of infection. Hence, establishing a relationship between the inoculation site (OI or GI) with disease progression and the mounting of *T. cruzi*-specific regional immune responses is an important issue to be considered. Here, we provide a discussion on studies performed in OI and GI in experimental models of acute infections, including *T. cruzi* infection.

## Introduction

Chagas disease, or American trypanosomiasis, caused by the hemoflagellate protozoan *Trypanosoma cruzi*, is a tropical neglected illness *Trypanosoma cruzi* (*T. cruzi*). Infection was initially enzooty and maintained among wild animals and insect vectors of the *Reduviidae* family. Deforestation in rural areas allowed vectors to invade human homes (1, 2).

Chagas disease transmission to humans can be classified in primary (vectorial, blood transfusion, congenital, and orally) and secondary (less frequent, such as laboratory accident, handling of infected animals, organ transplantation from infected donors, and hypothetically through sexual) routes of *T. cruzi* infection (3, 4). Different transmission routes present variable incubation period, such as oral, 3–22 days; vector feces near the bite, 4–15 days; blood transfusion, 8–120 days; and organ transplantation, 23–420 days (5–9). Besides the transmission pathway, mortality rates depend also on the patients’ clinical condition and on the time between disease diagnosis and beginning of treatment. Oral transmission results in a higher mortality, estimated between 8 and 35%, than the classical vector-borne infection (<5–10%) (5, 7–10).

From 1990 to 1993, the Brazilian Health Minister started to insert the Notifiable Diseases System of Information-SINAN (DATASUS) to control the number of acute cases in the country. Although underestimated, from 2002 to 2006 Brazil registered 2510 cases of acute Chagas disease according to the DATASUS system. Number of notifications decreased at the time that the pan-American Health Organization registered the interruption of *Triatoma infestans* population in the area in 2006 (11); however, numbers still reached 1,539 new cases in the DATASUS from 2007 to 2014. Nowadays, oral transmission of Chagas disease is the most frequent transmission route in the Brazilian Amazon region (12). Food/beverages contamination with *T. cruzi*-infected insect excreta, macerate, or reservoir meal is responsible for oral transmission in one to more than a hundred cases (outbreaks). It is noteworthy that oral transmission has been associated with high mortality and morbidity, including increased prevalence and severity of the cardiac pathology (myocarditis) (13–16). Argentina, Bolivia, Colombia, Ecuador, French Guiana, and Venezuela have also reported acute Chagas disease outbreaks associated with contaminated food consumption [revised in Ref. (11, 17)]. Fruits pasteurization is the appropriate pathway to kill the parasite, and it has been shown that outbreaks of oral infection in Brazilian Amazonia increase with seasonal months of higher açaí pulp production. Moreover, epidemiological data suggest that in the Pará state most of the cases are caused by consumption of artisanal açaí. Therefore, good practices of quality control could avoid the transmission, such as good agricultural practice and “bleaching” or “whitening” of the fruits (12, 18).

The infection is presently considered as a worldwide health problem with deficiencies in treatment, absence of vaccines, and world spreading (19–22).

## Parasite–Host Interaction and Target Tissues

*T. cruzi* presents one of the most complex life cycles among the trypanosomatids, alternating between vertebrate hosts, which comprises a wide range of mammals including humans and invertebrate hematophagous insects from the Reduviidae family (23, 24). Mammalian cell invasion by the *T. cruzi* is critical to its survival in the host. Once inside the vertebrate host, the metacyclic trypomastigotes are able to infect several nucleated mammalian cells at the inoculation site, such as macrophages, fibroblasts, epithelial cells, and others. The intracellular cycle in a mammalian cell presents different steps and begins at the moment that infective forms of *T. cruzi* interact with phagocytic or non-phagocytic surface molecules. These processes lead to cell signaling and internalization of the parasite through multiple endocytic pathways (25–27). *T. cruzi* proteins such as gp82, gp80, gp35/50, gp85, trans-sialidase, and host cell adhesion molecules such as mucins, VLA (very late antigen), and extracellular matrix proteins (ECM) such as laminin and fibronectin have been reported to contribute to parasite infection (23, 25, 28–33). In addition, *T. cruzi* proteases as cruzipain, oligopeptidase B, and Tc80 have been implicated in *T. cruzi* internalization (23, 31). In addition to presenting a large variety of surface molecules that can participate in host–parasite interaction, strain and forms (metacyclic trypomastigotes, tissue culture-derived trypomastigotes, and amastigotes) of the parasite differently express these molecules in the membrane. The capacity of trypomastigotes to interact with a diverse number of molecules on cell surface is determinant to improve invasion processes and allows the parasite to explore survival and multiplicative strategies in the host (23, 31, 34).

It is believed that any mammalian host cell class of molecules in the membrane are potential partners for *T. cruzi* recognition, and the expression of these molecules can vary depending on the cell type involved. Well-characterized groups of receptor are carbohydrates that contain galactosyl, mannosyl, and sialyl residues and lectin-like proteins (23, 26). Interestingly, *T. cruzi* is either able to use and increase expression of ECM in the host cell during the initial process of infection. Regarding *T. cruzi* surface molecules, it has been shown that trypomastigote forms present motifs that bind to cytokeratin 18, fibronectin, laminin, heparan sulfate proteoglycans, and integrins (35, 36).

The components involved in *T. cruzi* oral infection were suggested in experimental models. Hoft and colleagues demonstrated by histological analysis that after oral infection, *T. cruzi* invades and replicates inside epithelial cells within the gastric mucosa. This initial invasion is followed by the establishment of a progressive gastritis and further systemic dissemination of the parasite. Furthermore, hypertrophy and the presence of parasites in adjacent lymph nodes of stomach and inflammatory infiltrates in various organs (pancreas, liver, spleen, bone marrow, heart, duodenum, adrenal, brain, and/or skeletal muscle) were also described. Amastigote nests were detected in the gastric mucosal epithelium, but not in the upper gastrointestinal tract, like esophagus and oropharynx after oral infection. These data suggested that oral infection initiates in gastric mucosal followed by systemic dissemination (37).

Analysis of molecular mechanisms involved in *T. cruzi* interaction with host cells during oral infection is under investigation. It has been suggested that gastric epithelium express mucins that interacts with *T. cruzi* glycoproteins, such as gp82 and gp30, triggering a cascade of intracellular signaling in the parasite and at the host cell, leading to the mobilization of intracellular Ca^2+^ that is essential for parasite internalization (32, 34, 38, 39).

In line with this, previous studies of intrapharyngeal infection in mice, and *in vitro* studies of human epithelial cells have demonstrated the key role of glycoprotein gp82 during *T. cruzi* invasion in gastric of mucosal (40). Gp82 is present in metacyclic trypomastigotes forms, but not in amastigotes, epimastigotes, or tissue culture-derived trypomastigotes forms (41, 42). Interestingly, gp82 expressed in different *T. cruzi* strains is resistant to degradation by pepsin or proteinase K (43, 44). Metacyclic forms of *T. cruzi* recovered from the stomach 1 h after an intrapharyngeal inoculation in mice preserve the gp82 intact, and the parasite infectivity was not altered. Furthermore, *T. cruzi* gp82^−/−^ metacyclic forms have reduced gastric mucin-binding capacity, less efficient migration through the gastric mucin-coated and low infectivity in mice by the intrapharyngeal route when compared with metacyclic forms that express gp82 (44, 45).

GP30 is another glycoprotein involved in *T. cruzi* interaction with the gastric epithelium, binding the target cells in a receptor-dependent manner, inducing Ca^2+^ response and lysosome exocytosis, both required for the parasite internalization in the cell (44, 45). Interestingly, gp30 shows a lower affinity to gastric mucin-binding proteins as compared to gp82, and this seems to be associated with low infective capacity of gp82-deficient strains *in vivo* (45, 46). Different isolates of Y strain differ in the expression of gp82 and gp30 surface molecules and the ability to infect mice by the intragastric/intrapharyngeal inoculation (46).

The infection process is also influenced by gp90, a metacyclic stage-specific molecule, that binds to mammalian cells in receptor-dependent manner but, differently from gp82, this protein is unable to trigger Ca^2+^ signal and downmodulates the parasite cell invasion capacity (47). It has been shown that *T. cruzi* strains that express high levels of gp90 on the surface, in addition to gp82 and gp30, have low cellular infection capacity *in vitro*. However, recent *in vivo* studies indicated that infectivity of *T. cruzi* is also influenced by the susceptibility of g90 molecules to peptic digestion. *T. cruzi* strains expressing pepsin-resistant gp90 isoform show a low capacity to invade gastric mucosal epithelium after intrapharyngeal inoculation in mice, resulting in subpatent or low parasitemia. By contrast, *T. cruzi* strains expressing pepsin-susceptible gp90 produced high parasitemia and high mortality when given to mice by the intrapharyngeal route (43). In addition, analysis of extracellular vesicles and soluble proteins released by metacyclic trypomastigotes forms of *T. cruzi* has revealed presence of gp82 and gp90 surface molecules in these compartments (48, 49).

**Figure 1:**
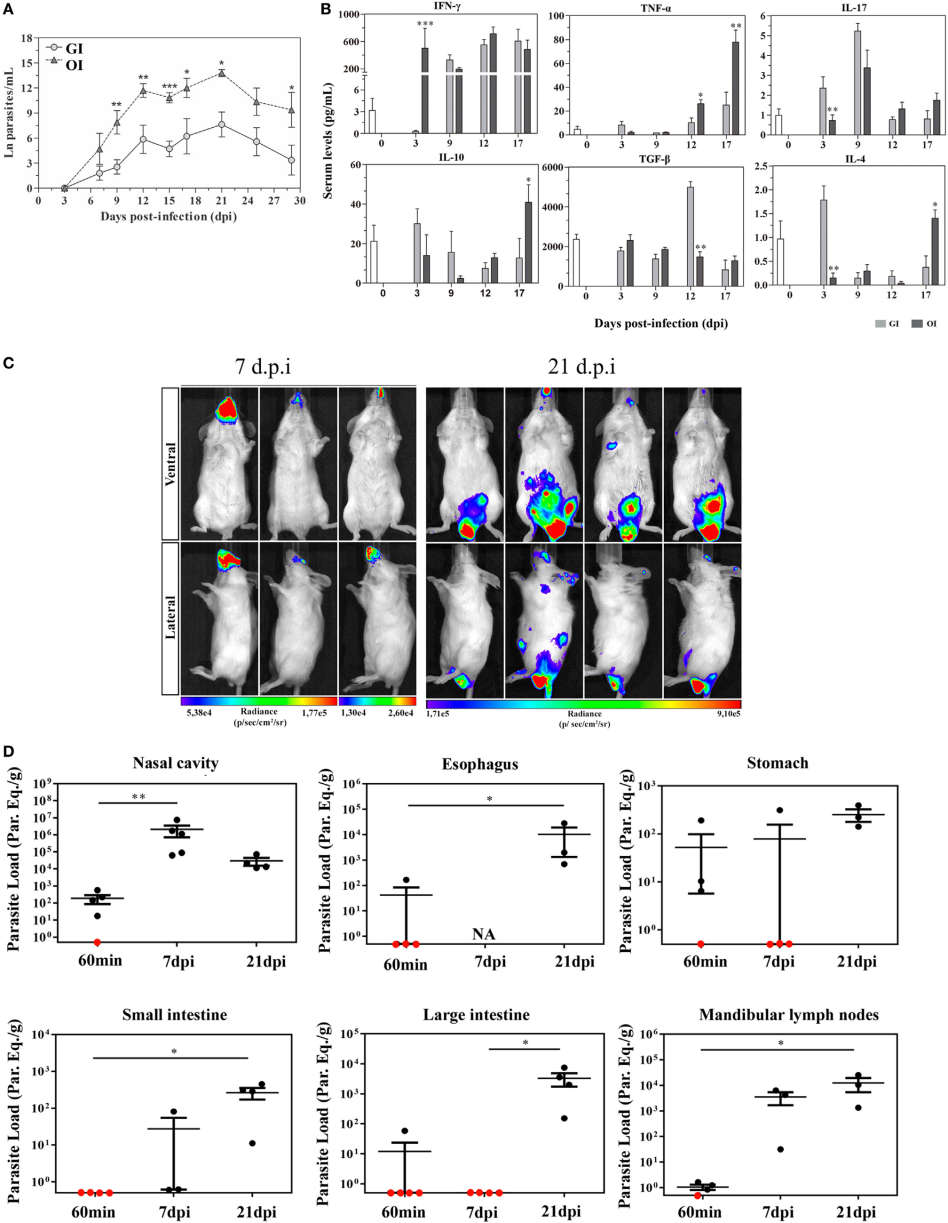
Severity and target tissues during acute phase of *Trypanosoma cruzi* orally infected mice. **(A)** Male BALB/c mice were infected with 5 × 10^4^ tissue culture-derived trypomastigotes forms of *T. cruzi* (Tulahuén strain) through gavage [gastrointestinal infection (GI)] or oral cavity (OI). Parasitemia (mean and SEM) was assessed during the acute phase and expressed as ln parasites per milliliter for statistical analysis. Parasites were counted by light microscopy, and parasitemia calculated by the Brenner method. Parasitemia comparisons were performed at different days post-infection (dpi), Kruskal–Wallis, Dunn’s post-test (until 15 dpi), and one-tailed Mann–Whitney (after 15 dpi) tests were used. **(A)** *n*: GI, 3 dpi = 7; 7 dpi = 22; 9 dpi = 29; 12 dpi = 17; 15 dpi = 45; 17 dpi = 10; 21 dpi = 24; 25 dpi = 16; 29 dpi = 11 and OI, 3 dpi = 4; 7 dpi = 9; 9 dpi = 14; 12 dpi = 22; 15 dpi = 40; 17 dpi = 12; 21 dpi = 14; 25 dpi = 8; 29 dpi = 6. Lower numbers represent early stages, when parasitemia was still undetectable and final stages, when mortality rates were too high. **(B)** Cytokine analysis in GI and OI mice. Male BALB/c mice were infected with 5 × 10^4^ tissue culture-derived trypomastigotes forms of *T. cruzi* (Tulahuén strain) through gavage (GI) or within oral cavity (OI). In the course of acute infection, serum was isolated and levels of cytokines (IFN-γ, TNF, IL-17, IL-10, and TGF-β) were quantified in uninfected control and infected mice by a multiplex analysis. The results are expressed as the mean values (±SEM) for each group/day post-infection. *n*: IFN-γ, uninfected (0) = 12; 3 dpi GI = 11, OI = 5; 9 dpi GI = 8, OI = 5; 12 dpi GI = 9, OI = 4; 17 dpi GI = 4, OI = 6. TNF, uninfected (0) = 11; 3 dpi GI = 10, OI = 10; 9, 12 dpi, GI = 3, OI = 3; 17 dpi, GI = 6, OI = 11. IL-17, uninfected (0) = 12; 3 dpi, GI = 10, OI = 10; 9 dpi, GI = 3, OI = 3; 12 dpi, GI = 5, OI = 5; 17 dpi, GI = 6, OI = 14. TGF-β, uninfected (0) = 6; 3 dpi, GI = 4, OI = 4; 9 dpi, GI = 5, OI = 5; 12 dpi, GI = 5, OI = 4; 17 dpi, GI = 2, OI = 5. IL-10 and IL-4, uninfected (0) = 6; 3, 9, 12 dpi, GI = 6, OI = 6; 17 dpi, GI = 3, OI = 8. Statistical analysis was performed using GraphPad Prism 5. *p = 0.05; **p = 0.01; ***p = 0.001. **(C)** Course of parasite distribution in oral infection. Male BALB/c mice were infected in the oral cavity (OI) with 1 × 10^6^ trypomastigotes forms of *T. cruzi* expressing luciferase (Dm28c-luc). Representative *in vivo* bioluminescence images were acquired in the same mice (*n* = 6), at 7 and 21 dpi, after 15 min of d-luciferin intraperitoneal administration (150 mg/kg), using IVIS^®^ Lumina image system (Xenogen). **(D)** *T. cruzi* loads in orally infected mice. Male BALB/c mice were infected in the oral cavity (OI) with 1 × 10^6^ trypomastigotes forms of *T. cruzi* expressing luciferase (Dm28c-luc). Organs and tissues were harvested for qPCR analysis to determine the parasite load (parasite equivalent/g) at 60 min, 7, and 21 dpi. The qPCR was performed in multiplex, targeting *T. cruzi* nuclear satellite DNA (Sat DNA) and IAC (internal amplification control), as a quality control. Parasite load in the nasal cavity (*n*: 60 min and 7 dpi = 5; 21 dpi = 4), esophagus (*n*: 60 min = 4; 21 dpi = 3), stomach (*n*: 60 min and 7 dpi = 4; 21 dpi = 3), small intestine (*n*: 60 min = 5; 7 dpi = 3; 21 dpi = 4), large intestine (*n*: 60 min = 5; 7 and 21 dpi = 4), and mandibular lymph nodes (*n*: 60 min = 4; 7 and 21 dpi = 3). Red dots: no parasite detection. Values present mean ± SEM. Kruskal–Wallis (Dunn’s post-test) for group kinetics. Statistical analysis was performed using Graph Pad Prism 5. *p < 0.05, **p < 0.01. Adapted from Barreto-de-Albuquerque et al. (51) and Silva-dos Santos et al. (52).

A variety number of molecules involved in parasite–host cell interactions are potential candidates in oral infection. During the oral infection, parasites come across different cells throughout the gastrointestinal tract, from tissues as the mouth to intestines. In previous data, Diaz-Ungría and Bracho showed signs of a possible *T. cruzi* penetration in the oral, esophageal, gastric, and intestinal mucosa with local eosinophilia, infiltrated lymphocytes and monocytes in histological sections from dogs after oral infection (50). We have recently demonstrated that the site of parasite entrance, through the mouth (oral infection—OI), which is more similar to natural infection, versus gastrointestinal infection (GI) promotes different host immune response and mortality. Thus, compared with GI mice, OI mice presented elevated infection rate, parasitemia, and higher Th1 cytokines (51) (Figures 1A,B). This distinct immunological response and infection severity according to the different mucosal pathways highlighted important considerations concerning the primary site of *T. cruzi* infection in the oral route and indicated that the pathophysiology in this model may not be the same when parasites are administrated into the oral cavity or by gavage into the stomach (intrapharyngeal/intragastric).

In a recent study, the site of parasite entry in OI mice, inoculating *T. cruzi* directly in the mouth and analyzing by bioluminescence imaging corroborates the hypothesis that oral cavity is a potential critical site of initial *T. cruzi* infection before spreading to other organs in the acute phase. Moreover, OI leads to *T. cruzi* entrance in the palate, multiplication at the nasal cavity and dissemination to central nervous system and peripheral tissues. These evidences suggest that oral cavity is the primary site of infection and the nasal cavity comprises most of the parasite replication (52) (Figures 1C,D). Interestingly, facial edema and paresthesia of the tongue were already described in patients infected with *T. cruzi* by the oral route (53).

The mouth/oral cavity is also a target tissue for different viral, bacterial, and fungal infections disease, such as Herpes virus type 1 and 2, *Helicobacter pylori, Candida albicans*, and others disease (54–56). The oral cavity contains distinct mucosal surfaces and molecules expression, such as mucins, in which microorganisms can bind and, consequently colonize this anatomical region (57). The oral mucosa is coated by a film of mucus consisting of lipids, glycosylated proteins, such as mucin immunoglobulins, as well as growth factors and others. The mucins are considered as the first line of defense in the oral cavity, preventing the attachment of certain pathogens to the epithelium or forming aggregates facilitating the elimination of pathogens by the organism. However, some pathogens can bind in the carbohydrate structures present in the mucins, such as sialic acids, which favors access to epithelial cells and cell invasion (57–59).

Previous data demonstrated that *Streptococcus* sp. binds to salivary mucins on the surface of the tooth, being one of the first steps in the formation of dental plaque (60). Studies using *Tannerella forsythia*, one of the major bacterial pathogens associated with periodontitis, uncovered that glycoprotein-associated sialic acid in terminal sugars on the surface of oral cavity epithelium is important for the adhesion and invasion of these bacteria. In this study, parasite inactivation by mutation or inhibition of NanH sialidase decreased the adhesion and invasion of *T. forsythia* in human gingival epithelial cell culture lines (OBA-9). The NanH sialidase activity is specific for α-2,3 sialic acid present on the surface of gingival epithelial cells, suggesting its role in parasite adhesion and invasion (61, 62).

In line with these findings, Lakdawala and colleagues demonstrated that the soft palate is a relevant focus of influenza viruses’ infection. The soft palate is a mucin-rich environment, which favors the infection and may contribute to airborne transmission. Furthermore, the expression of α-2,3 sialic acids, the viral hemagglutinin ligands, is detected on the soft palate, in the regions of the oral surface and the nasopharyngeal tissues from humans and ferret (63).

Interestingly, α-2,3 sialic acids are the main molecule involved in *T. cruzi* trans-sialidase-mediated binding. Trans-sialidase are considered as an important virulence factor, since this enzyme is able to reduce host cell immune response and mediates *T. cruzi* and host cells adhesion (33). It has been shown that trans-sialidase binds to host sialoglycans, generating “eat me” signals in epithelial cells, which facilitates parasite entry into non-phagocytic cells (64). Notably, the mouth seems to be a potential source of infection and this knowledge contributes to the elucidation of the target tissue/organs and the molecular components regulating the establishment of *T. cruzi* oral infection and its pathogenesis.

## Immune Response and Disease Outcome in Experimental Models

The most widely used experimental model to study *T. cruzi* infection has been for years the intraperitoneal (IP) inoculation of the parasite in mice, in which 10^2^ trypomastigotes are able to promote functional alterations in the immune system from 14 days post-infection (dpi) (65). However, this pathway does not mimic the natural infection through contaminated excreta left by the vector after biting. More importantly, especially in Brazil and other endemic countries, the most frequent transmission route has been reported to be by ingestion of contaminated food and beverages (7, 17, 66, 67). Several approaches to address oral infection in mice have been described in the literature, such as intrapharyngeal, intragastric, and in the oral cavity inoculation (37, 44, 45, 51, 68–70).

Comparing mucosal routes through the digestive tube with systemic inoculation, differences in disease outcome and immune response can be observed. Intraperitoneally infected mice present higher parasitemia and mortality than intragastric or oral cavity-inoculated mice with the same inoculum (51, 69, 71). Besides, IP-infected mice also start to die earlier than GI/OI-infected and present 80–100% mortality, while GI/OI results in higher survival rates. Still, OI leads to parasitemia and mortality levels higher than in GI models. Infection through gavage (intragastrically) presents less percentage of mice with patent parasitemia, parasitemia, and mortality than IP injection (51, 70). Despite intermediate parasitemia and mortality levels between GI and IP, OI infection leads to a percentage of mice with patent parasitemia similar to IP (49.3% for GI and 97.5% for OI) (51). These temporal and quantitative differences in parasitemia might be related to the distinct barriers the parasite needs to cross after these inoculation routes. As it has been discussed in the literature, the route of parasite inoculation affects the pathogenesis and disease outcome of experimental *T. cruzi* infection (72).

After oral infection, parasites have been detected in several tissues, and even where they are not detected, inflammatory infiltrates are found (37, 52). Systemic *versus* mucosal *T. cruzi* infection leads to distinct disease patterns. Systemic infections with Peruvian strain, such as IP, IV (intravenous), or SC (subcutaneous) promote higher infection rates (67–100%) and mortality than mucosal, such as OI, GI, intrarectal, genitalia, or conjunctival infection (17–67%) (73). By contrast, the study by Caradonna and Pereiraperrin (74), mice infected with Tulahuén strain through intranasal (IN) route present higher mortality than SC. In addition, after an oral inoculation (oropharynx), insect-derived metacyclic trypomastigotes are more infective when compared to cutaneous challenge (over puncture wound that is not the same as the SC) (75).

Inoculation route can also lead to preferential tropism, as well as distinct local and systemic immune responses (51, 52, 72, 74) (Table 1). Inflammatory infiltrates can be found in the heart and the severity is not necessarily the main cause of death (37, 51, 69). Infiltration of immune cells is observed in several organs regardless the presence of parasite (37). The literature shows that Tulahuén strain of *T. cruzi* induces TNF production and apoptosis of hepatocytes (76). In this regard, OI and GI infection leads to apoptosis in the liver and in the heart of acute infected mice and the macrophages are the main source of TNF. These different pathways can also lead to elevated serum IFN-γ levels and TNF, especially in OI (51). Also in the heart higher levels of TNF mRNA is detected in OI when compared with GI. This elevated TNF levels in OI may be associated with cardiac, spleen, and hepatic damage, as well as toxic shock in mice, as reported in studies with other models (51, 77, 78). Besides, it can be considered one of the factors for death in mice, since blockage of this cytokine improves the survival (51).

**Table 1:** Cytokines are differentially expressed according to the infection route.

	GI	Reference	OI	Reference
CCL3	↑ stomach (a; c)/spleen (c)	(79)	N.A.	–
CCL5	↑ stomach (c)/heart (c)	(79)	N.A.	–
CXCL1	↑ stomach (c)/heart (c)	(79)	N.A.	–
CXCL10	↑ stomach (c)/heart (c)/spleen (c)	(79)	N.A.	–
CXCL9	↑ stomach (c)/heart (c)/spleen (c)	(79)	N.A.	–
G-CSF	↑ stomach (c)	(79)	N.A.	–
IFN-γ	↑ stomach (a; c)/spleen (c)/serum (a)	(51, 79)	↑ IEL (a)/LP (a)/↑↑ serum (a)	(37, 51)
IL-10	↑ stomach (a; c)/heart (c)/spleen (a)	(79)	↑ serum (a)	(51)
IL-12	↑ stomach (a; c)/heart (c)/spleen (a)	(79)	N.A.	
IL-17	↑ serum (a)	(51, 79)	↑↑ serum (a)	(51)
IL-2	↑ stomach (c)/spleen (c)	(79)	N.A.	–
IL-3	↑ stomach (a; c)	(79)	N.A.	–
IL-4	↑ stomach (a; c)/spleen (c)	(79)	↑ serum (a)	(51)
IL-6	↑ spleen (c)	(79)	N.A.	–
IL-7	↑ stomach (c)/spleen (c)	(79)	N.A.	–
IL-9	↑ spleen (c)	(79)	N.A.	–
M-CSF	↑ stomach (c)	(79)	N.A.	–
TGF-β	↑↑ serum (a)	(51)	↑ serum (a)	(51)
TNF	↑ stomach (c)/spleen (c)/liver (a)/heart (a)/↑ serum (a)	(51, 79)	↑ liver/heart/↑↑ serum (a)	(51)
GM-CSF	↑ spleen (a; c)	(79)	N.A.	–

*GI, gastrointestinal infection; OI, oral infection; a, acute phase; c, chronic phase; N.A., not analyzed; IEL, intraephitelial lymphocytes; LP, lamina propria*.

After OI or GI, different cell types can be found within the heart and liver, such as CD4^+^ and CD8^+^ cells, neutrophils, and macrophages. Among them, macrophages constitute the main source of tissue TNF (51). In acute and chronic phase, inflammation can be detected in the stomach and heart after GI infection, followed by alterations in cytokine production. An increase of IL-12, IFN-γ, IL-4, IL-10, CCL3/4, and IL-3 is observed in the stomach during the acute phase of the disease and IL-12, TNF-α, CCL3/4, CXCL1, CCL5, CXCL9, CXCL10, G-CSF, M-CSF, IL-2, and IL-7 in the chronic phase. Hoft and collaborators demonstrated that after oral inoculation, *T. cruzi* infection within the gastric epithelium is able to stimulate B cell responses with parasite-specific IgA and IgG, suggesting activation of these cells in mucosal inductive sites, such as Peyer’s patches, although the presence of parasite was not proven there. Furthermore, gastric intraepithelial lymphocytes and from lamina propria produce IFN-γ (37). In the heart, IL-10 and CXCL1 increase in animals GI-infected with the CL strain, in addition to IL-12, IL-10, CXCL9, and CXCL10 during the chronic phase. Of note, this profile can vary according to strain (79). Yet, little is known after oral inoculation of the parasite.

It was already described after IP infection, alterations in secondary lymphoid organs are observed in acute infection with an increase in total cell numbers and individual subsets as well as cytokine production in the subcutaneous lymph nodes and spleen, and a decrease in mesenteric lymph nodes and thymus (65, 80). After GI, there is an increase of neutrophils, lymphocytes, and monocytes and a reduction of the number of eosinophils in GI- and IP-infected mice. As demonstrated in Domingues and colleagues study, the peak of parasitemia in GI at 18 dpi is correlated with an increase in monocytes in the blood. The spleen also increases in GI, mainly CD8^+^ cells and double-positive CD8^+^CD4^+^, but at a later time and the thymus is slightly increased instead of the atrophy observed after IP (70). High levels of IL-12, IL-10, and GM-CSF are expressed in the spleen during the acute phase of CL strain-infected mice, while IFN-γ, TNF-α, IL-6, IL-4, IL-9, CCL3/4, CXCL9, CXCL10, GM-CSF, IL-2, and IL-7 are elevated during chronic infection (79). The mesenteric lymph nodes decrease in GI with reduction of CD4^+^ cells (70). Of note, the only study addressing lymphoid organ alteration after oral infection (oral cavity inoculation) reports an increase of gastric lymph nodes (37).

Regarding systemic cytokines GI and OI mice have a high concentration of serum IL-4, while OI leads to lower amounts of the regulatory cytokine IL-10 and TGF-β. These cytokines are known to inhibit macrophage microbicidal function, protecting the host from tissue damage (77, 81). Furthermore, inoculation of parasite through digestive mucosa (oral and more in GI) triggers IL-17 production, which is reflected in the serum. IL-17-producing cells have also been described to contribute to the formation of the gastrointestinal barrier (82). Moreover, mucosal infections (IN or OI/GI) with bacteria, such as *Listeria monocytogenes* (Lm), *Streptococcus pyogenes*, and *Francisella tularensis* leads to Th17 responses, while the systemic routes (IV or SC) trigger a Th1 response (83).

For different infection models systemic inoculations, IP, IV, and SC have been widely used. Although these approaches do not always necessarily mimic the natural transmission pathway. Our group and others have already demonstrated that the route of parasite administration is relevant for the disease outcome in infections by different pathogens (72, 84). Besides as ideal experimental model should mimic all phases of infection, including the transmission pathway (84). In this regard, for food-borne diseases, oral inoculation is an essential issue to consider.

Considering the human counterpart in Chagas disease, oral transmission has become more epidemiologically relevant and the outbreaks are related to contaminated food ingestion (85). Interestingly, facial edema is frequently observed in these patients (53). In experimental models, we described that host response is distinct when parasites are delivered into the oral cavity or by gavage (51). As it has been discussed also in non-infectious models, the oral cavity represents the first contact with the organism after ingestion and presents an underexplored environment. Thus, it should really be considered as more than just the entrance for the gastrointestinal tract (86). Tolerogenic dendritic cells producing IL-10 and IL-12 (regulatory and inflammatory profiles) can capture parasite/antigens within the mucosa in the oral cavity and in the gastrointestinal tract from where they can also be drained to the liver by the portal system (87, 88). Thus, regarding oral infections, parasite delivery into the oral cavity or by gavage (intrapharyngeal/gastrointestinal) should not be assumed as equivalent processes.

The importance to standardize the nomenclature and the choice among different approaches to address “oral” infection, such as *ad libitum*, oral gavage and in the oral cavity has been discussed also in the context of other food-borne diseases, such as Listeriosis, caused by the bacteria Lm (84). After GI Lm inoculation, high amounts of Lm and specific T cells are found in the intestinal mucosa, mesenteric lymph nodes, spleen, and liver, whereas ingestion of Lm-contaminated bread promotes increased and phenotypically distinct intestinal resident memory cells (T_RM_) compared with other routes of infection (89–91). Moreover, IV and IN routes are able to induce T_H_1 and T_H_17 CD4^+^ cells, respectively, but T_H_1 cells from IV were are more likely to originate a memory cell pool than T_H_17 from IN (92).

## Conclusion and Perspectives

Nowadays, *T. cruzi* oral transmission is an important route of infection in Latin American countries. Despite its relevance, significant studies about this form of parasite infection are largely lacking. Experimental studies related to oral *T. cruzi* and other infective agents usually comprise inoculation in the mouth (OI) or intragastrically/intrapharyngeal (GI), being roughly considered as similar routes of infection. In this review, we unraveled the intrinsic importance of specific (and distinct) tissues involved in the primary site of an infective agent entrance, resulting in regional immune response and differential disease outcome. New studies investigating the influence of target tissues and host–parasite interactions in OI and GI must be performed.

## Author Contributions

JM, JBA, DSS, and JS contributed with text writing and review construction.

## Conflict of Interest Statement

The authors declare that the research was conducted in the absence of any commercial or financial relationships that could be construed as a potential conflict of interest.

## References

[1] Schofield CJ Diotaiuti L Dujardin JP The process of domestication in Triatominae. Mem Inst Oswaldo Cruz (1999) 94(Suppl 1):375–8. 10.1590/S0074-02761999000700073 10677759

[2] Coura JR Vinas PA Junqueira AC. Ecoepidemiology, short history and control of Chagas disease in the endemic countries and the new challenge for non-endemic countries. Mem Inst Oswaldo Cruz (2014) 109(7):856–62. 10.1590/0074-0276140236 25410988 PMC4296489

[3] Dorn P Buekens P Hanford E Whac-a-mole: future trends in Chagas transmission and the importance of a global perspective on disease control. Future Microbiol (2007) 2:365–7. 10.2217/17460913.2.4.365 17683271

[4] Tarleton RL Reithinger R Urbina JA Kitron U Gurtler RE The challenges of Chagas disease – grim outlook or glimmer of hope. PLoS Med (2007) 4:e332 10.1371/journal.pmed.0040332 18162039 PMC2222930

[5] Wendel S. Transfusion transmitted Chagas disease: is it really under control? Acta Trop (2010) 115:28–34. 10.1016/j.actatropica.2009.12.006 20044970

[6] Bern C Kjos S Yabsley MJ Montgomery SP *Trypanosoma cruzi* and Chagas’ disease in the United States. Clin Microbiol Rev (2011) 24:655–81. 10.1128/CMR.00005-11 21976603 PMC3194829

[7] Shikanai-Yasuda MA Carvalho NB. Oral transmission of Chagas disease. Clin Infect Dis (2012) 54:845–52. 10.1093/cid/cir956 22238161

[8] Noya BA Diaz-Bello Z Colmenares C Ruiz-Guevara R Mauriello L Munoz-Calderon A Update on oral Chagas disease outbreaks in Venezuela: epidemiological, clinical and diagnostic approaches. Mem Inst Oswaldo Cruz (2015) 110:377–86. 10.1590/0074-02760140285 25946155 PMC4489475

[9] Alarcón de Noya B Noya González O Robertson LJ *Trypanosoma cruzi* as a foodborne pathogen. SpringerBriefs in Food, Health, and Nutrition. New York, Heidelberg, Dordrecht, London: Springer (2016). 92 p.

[10] Rassi A Jr Rassi A Marin-Neto JA. Chagas disease. Lancet (2010) 375:1388–402. 10.1016/S0140-6736(10)60061-X 20399979

[11] Sanchez LV Ramirez JD Congenital and oral transmission of American trypanosomiasis: an overview of physiopathogenic aspects. Parasitology (2013) 140:147–59. 10.1017/S0031182012001394 23010131

[12] Santos V Meis J Savino W Andrade JAA Vieira J Coura JR Acute Chagas disease in the state of para, Amazon region: is it increasing? Mem Inst Oswaldo Cruz (2018) 113:e170298 10.1590/0074-02760170298 29742200 PMC5951676

[13] Barbosa-Ferreira JM Nobre AF Maldonado JG Borges-Pereira J Zauza PL Coura JR [Stroke in a chronic autochthonous chagasic patient from the Brazilian Amazon]. Rev Soc Bras Med Trop (2010) 43:751–3. 10.1590/S0037-86822010000600034 21181041

[14] Pinto AY Harada GS Valente V Abud JE Gomes F Souza GC [Cardiac attacks in patients with acute Chagas disease in a family micro-outbreak, in Abaetetuba, Brazilian Amazon]. Rev Soc Bras Med Trop (2001) 34:413–9. 10.1590/S0037-86822001000500003 11600906

[15] Vinas Albajar P Laredo SV Terrazas MB Coura JR [Dilated cardiomyopathy in patients with chronic chagasic infection: report of two fatal autochthonous cases from Rio Negro, state of Amazonas, Brazil]. Rev Soc Bras Med Trop (2003) 36:401–7. 10.1590/S0037-86822003000300013 12908042

[16] Ianni BM Mady C [The sugarcane juice was delicious, but…]. Arq Bras Cardiol (2005) 85:379–81. 10.1590/S0066-782X2005001900001 16429196

[17] de Noya BA Gonzalez ON. An ecological overview on the factors that drives to *Trypanosoma cruzi* oral transmission. Acta Trop (2015) 151:94–102. 10.1016/j.actatropica.2015.06.004 26066984

[18] Barbosa RL Pereira KS Dias VL Schmidt FL Alves DP Guaraldo AM Virulence of *Trypanosoma cruzi* in Acai (*Euterpe oleraceae* Martius) pulp following mild heat treatment. J Food Prot (2016) 79:1807–12. 10.4315/0362-028X.JFP-15-595 28221851

[19] Hotez PJ Molyneux DH Stillwaggon E Bentwich Z Kumaresan J Neglected tropical diseases and HIV/AIDS. Lancet (2006) 368:1865–6. 10.1016/S0140-6736(06)69764-X 17126708

[20] Savino W Villa-Verde DM Mendes-Da-Cruz DA Silva-Monteiro E Perez AR Aoki Mdel P Cytokines and cell adhesion receptors in the regulation of immunity to *Trypanosoma cruzi*. Cytokine Growth Factor Rev (2007) 18:107–24. 10.1016/j.cytogfr.2007.01.010 17339126

[21] Malik LH Singh GD Amsterdam EA Chagas heart disease: an update. Am J Med (2015) 128(1251):e1257–9. 10.1016/j.amjmed.2015.04.036 26052027

[22] Thakare R Dasgupta A Chopra S. An update on benznidazole for the treatment of patients with Chagas disease. Drugs Today (Barc) (2018) 54:15–23. 10.1358/dot.2018.54.1.2753402 29569658

[23] de Souza W de Carvalho TM Barrias ES Review on *Trypanosoma cruzi*: host cell interaction. Int J Cell Biol (2010) 2010:295394 10.1155/2010/295394 20811486 PMC2926652

[24] Coura JR Junqueira AC Ecological diversity of *Trypanosoma cruzi* transmission in the Amazon basin. The main scenaries in the Brazilian Amazon. Acta Trop (2015) 151:51–7. 10.1016/j.actatropica.2015.04.029 26254002

[25] Maeda FY Cortez C Yoshida N. Cell signaling during *Trypanosoma cruzi* invasion. Front Immunol (2012) 3:361. 10.3389/fimmu.2012.00361 23230440 PMC3515895

[26] Barrias ES de Carvalho TM De Souza W. *Trypanosoma cruzi*: entry into mammalian host cells and parasitophorous vacuole formation. Front Immunol (2013) 4:186. 10.3389/fimmu.2013.00186 23914186 PMC3730053

[27] Cardoso MS Reis-Cunha JL Bartholomeu DC Evasion of the immune response by *Trypanosoma cruzi* during acute infection. Front Immunol (2015) 6:659 10.3389/fimmu.2015.00659 26834737 PMC4716143

[28] Schenkman S Jiang MS Hart GW Nussenzweig V. A novel cell surface trans-sialidase of *Trypanosoma cruzi* generates a stage-specific epitope required for invasion of mammalian cells. Cell (1991) 65:1117–25. 10.1016/0092-8674(91)90008-M 1712251

[29] Buscaglia CA Campo VA Frasch AC Di Noia JM. *Trypanosoma cruzi* surface mucins: host-dependent coat diversity. Nat Rev Microbiol (2006) 4:229–36. 10.1038/nrmicro1351 16489349

[30] Yoshida N Cortez M. *Trypanosoma cruzi*: parasite and host cell signaling during the invasion process. Subcell Biochem (2008) 47:82–91. 10.1007/978-0-387-78267-6_6 18512343

[31] Villalta F Scharfstein J Ashton AW Tyler KM Guan F Mukherjee S Perspectives on the *Trypanosoma cruzi*-host cell receptor interactions. Parasitol Res (2009) 104:1251–60. 10.1007/s00436-009-1383-3 19283409 PMC2696482

[32] Yoshida N. Molecular mechanisms of *Trypanosoma cruzi* infection by oral route. Mem Inst Oswaldo Cruz (2009) 104(Suppl 1):101–7. 10.1590/S0074-02762009000900015 19753464

[33] Freire-de-Lima L Fonseca LM Oeltmann T Mendonca-Previato L Previato JO. The trans-sialidase, the major *Trypanosoma cruzi* virulence factor: three decades of studies. Glycobiology (2015) 25:1142–9. 10.1093/glycob/cwv057 26224786

[34] Yoshida N. *Trypanosoma cruzi* infection by oral route: how the interplay between parasite and host components modulates infectivity. Parasitol Int (2008) 57:105–9. 10.1016/j.parint.2007.12.008 18234547

[35] Ortega-Barria E Pereira ME. A novel *T. cruzi* heparin-binding protein promotes fibroblast adhesion and penetration of engineered bacteria and trypanosomes into mammalian cells. Cell (1991) 67:411–21. 10.1016/0092-8674(91)90192-2 1655283

[36] Calvet CM Melo TG Garzoni LR Oliveira FO Jr Neto DT Meirelles MNSL Current understanding of the *Trypanosoma cruzi*-cardiomyocyte interaction. Front Immunol (2012) 3:327 10.3389/fimmu.2012.00327 23115558 PMC3483718

[37] Hoft DF Farrar PL Kratz-Owens K Shaffer D. Gastric invasion by *Trypanosoma cruzi* and induction of protective mucosal immune responses. Infect Immun (1996) 64:3800–10. 8751932 10.1128/iai.64.9.3800-3810.1996 PMC174296

[38] Staquicini DI Martins RM Macedo S Sasso GR Atayde VD Juliano MA Role of GP82 in the selective binding to gastric mucin during oral infection with *Trypanosoma cruzi*. PLoS Negl Trop Dis (2010) 4:e613. 10.1371/journal.pntd.0000613 20209152 PMC2830468

[39] Yoshida N Tyler KM Llewellyn MS. Invasion mechanisms among emerging food-borne protozoan parasites. Trends Parasitol (2011) 27:459–66. 10.1016/j.pt.2011.06.006 21840261

[40] Neira I Silva FA Cortez M Yoshida N. Involvement of *Trypanosoma cruzi* metacyclic trypomastigote surface molecule gp82 in adhesion to gastric mucin and invasion of epithelial cells. Infect Immun (2003) 71:557–61. 10.1128/IAI.71.1.557-561.2003 12496211 PMC143373

[41] Teixeira MM Yoshida N. Stage-specific surface antigens of metacyclic trypomastigotes of *Trypanosoma cruzi* identified by monoclonal antibodies. Mol Biochem Parasitol (1986) 18:271–82. 10.1016/0166-6851(86)90085-X 3515178

[42] Araya JE Cano MI Yoshida N Da Silveira JF. Cloning and characterization of a gene for the stage-specific 82-kDa surface antigen of metacyclic trypomastigotes of *Trypanosoma cruzi*. Mol Biochem Parasitol (1994) 65:161–9. 10.1016/0166-6851(94)90124-4 7935622

[43] Cortez M Silva MR Neira I Ferreira D Sasso GR Luquetti AO *Trypanosoma cruzi* surface molecule gp90 downregulates invasion of gastric mucosal epithelium in orally infected mice. Microbes Infect (2006) 8:36–44. 10.1016/j.micinf.2005.05.016 16153873

[44] Cortez C Yoshida N Bahia D Sobreira TJ Structural basis of the interaction of a *Trypanosoma cruzi* surface molecule implicated in oral infection with host cells and gastric mucin. PLoS One (2012) 7:e42153 10.1371/journal.pone.0042153 22860068 PMC3409152

[45] Cortez M Neira I Ferreira D Luquetti AO Rassi A Atayde VD Infection by *Trypanosoma cruzi* metacyclic forms deficient in gp82 but expressing a related surface molecule, gp30. Infect Immun (2003) 71:6184–91. 10.1128/IAI.71.11.6184-6191.2003 14573635 PMC219548

[46] Cortez C Martins RM Alves RM Silva RC Bilches LC Macedo S Differential infectivity by the oral route of *Trypanosoma cruzi* lineages derived from Y strain. PLoS Negl Trop Dis (2012) 6:e1804 10.1371/journal.pntd.0001804 23056658 PMC3464286

[47] Malaga S Yoshida N. Targeted reduction in expression of *Trypanosoma cruzi* surface glycoprotein gp90 increases parasite infectivity. Infect Immun (2001) 69:353–9. 10.1128/IAI.69.1.353-359.2001 11119524 PMC97890

[48] Bayer-Santos E Cunha-E-Silva NL Yoshida N Franco Da Silveira J. Expression and cellular trafficking of GP82 and GP90 glycoproteins during *Trypanosoma cruzi* metacyclogenesis. Parasit Vectors (2013) 6:127. 10.1186/1756-3305-6-127 23634710 PMC3652755

[49] Clemente TM Cortez C Novaes Ada S Yoshida N. Surface molecules released by *Trypanosoma cruzi* metacyclic forms downregulate host cell invasion. PLoS Negl Trop Dis (2016) 10:e0004883. 10.1371/journal.pntd.0004883 27483135 PMC4970754

[50] Diaz-Ungría C Bracho JS Camino que sigue el *Trypanosoma cruzi* en perros infectados por vía bucal: nuevos estudios. Rev Vet Venezuelana (1970) 44:114–9.

[51] Barreto-de-Albuquerque J Silva-dos-Santos D Perez AR Berbert LR de Santana-Van-Vliet E Farias-de-Oliveira DA *Trypanosoma cruzi* infection through the oral route promotes a severe infection in mice: new disease form from an old infection? PLoS Negl Trop Dis (2015) 9:e0003849. 10.1371/journal.pntd.0003849 26090667 PMC4474863

[52] Silva-dos-Santos D Barreto-de-Albuquerque J Guerra B Moreira OC Berbert LR Ramos MT Unraveling Chagas disease transmission through the oral route: gateways to *Trypanosoma cruzi* infection and target tissues. PLoS Negl Trop Dis (2017) 11:e0005507 10.1371/journal.pntd.0005507 28379959 PMC5397068

[53] Pinto AY Ferreira AG Jr Valente Vda C Harada GS Valente SA. Urban outbreak of acute Chagas disease in Amazon region of Brazil: four-year follow-up after treatment with benznidazole. Rev Panam Salud Publica (2009) 25:77–83. 10.1590/S1020-49892009000100012 19341528

[54] Stoopler ET Sollecito TP Oral mucosal diseases: evaluation and management. Med Clin North Am (2014) 98:1323–52. 10.1016/j.mcna.2014.08.006 25443679

[55] Salvatori O Puri S Tati S Edgerton M. Innate immunity and saliva in *Candida albicans*-mediated oral diseases. J Dent Res (2016) 95:365–71. 10.1177/0022034515625222 26747422 PMC4802782

[56] Yee JK. Helicobacter pylori colonization of the oral cavity: a milestone discovery. World J Gastroenterol (2016) 22:641–8. 10.3748/wjg.v22.i2.641 26811613 PMC4716065

[57] Wu RQ Zhang DF Tu E Chen QM Chen W. The mucosal immune system in the oral cavity—an orchestra of T cell diversity. Int J Oral Sci (2014) 6:125–32. 10.1038/ijos.2014.48 25105816 PMC4170154

[58] Tabak LA In defense of the oral cavity: structure, biosynthesis, and function of salivary mucins. Annu Rev Physiol (1995) 57:547–64. 10.1146/annurev.ph.57.030195.002555 7778877

[59] Derrien M van Passel MW van de Bovenkamp JH Schipper RG de Vos WM Dekker J. Mucin-bacterial interactions in the human oral cavity and digestive tract. Gut Microbes (2010) 1:254–68. 10.4161/gmic.1.4.12778 21327032 PMC3023607

[60] Murray PA Prakobphol A Lee T Hoover CI Fisher SJ. Adherence of oral streptococci to salivary glycoproteins. Infect Immun (1992) 60:31–8. 1729194 10.1128/iai.60.1.31-38.1992 PMC257499

[61] Honma K Mishima E Sharma A. Role of *Tannerella forsythia* NanH sialidase in epithelial cell attachment. Infect Immun (2011) 79:393–401. 10.1128/IAI.00629-10 21078857 PMC3019913

[62] Roy S Honma K Douglas CW Sharma A Stafford GP. Role of sialidase in glycoprotein utilization by *Tannerella forsythia*. Microbiology (2011) 157:3195–202. 10.1099/mic.0.052498-0 21885482 PMC3352272

[63] Lakdawala SS Jayaraman A Halpin RA Lamirande EW Shih AR Stockwell TB The soft palate is an important site of adaptation for transmissible influenza viruses. Nature (2015) 526:122–5. 10.1038/nature15379 26416728 PMC4592815

[64] Butler CE de Carvalho TM Grisard EC Field RA Tyler KM. Trans-sialidase stimulates eat me response from epithelial cells. Traffic (2013) 14:853–69. 10.1111/tra.12078 23601193 PMC3770925

[65] de Meis J Morrot A Farias-de-Oliveira DA Villa-Verde DM Savino W. Differential regional immune response in Chagas disease. PLoS Negl Trop Dis (2009) 3:e417. 10.1371/journal.pntd.0000417 19582140 PMC2700264

[66] Benchimol Barbosa PR. The oral transmission of Chagas’ disease: an acute form of infection responsible for regional outbreaks. Int J Cardiol (2006) 112:132–3. 10.1016/j.ijcard.2005.11.087 16600406

[67] Dias JC. [Notes about of *Trypanosoma cruzi* and yours bio-ecology characteristics with agents of the transmission by meals]. Rev Soc Bras Med Trop (2006) 39:370–5. 10.1590/S0037-86822006000400010 17119753

[68] Kirchhoff LV Hoft DF. Immunization and challenge of mice with insect-derived metacyclic trypomastigotes of *Trypanosoma cruzi*. Parasite Immunol (1990) 12:65–74. 10.1111/j.1365-3024.1990.tb00936.x 2107500

[69] Camandaroba EL Pinheiro Lima CM Andrade SG. Oral transmission of Chagas disease: importance of *Trypanosoma cruzi* biodeme in the intragastric experimental infection. Rev Inst Med Trop Sao Paulo (2002) 44:97–103. 10.1590/S0036-46652002000200008 12048547

[70] Domingues CS Hardoim DJ Souza CS Cardoso FO Mendes VG Previtalli-Silva H Oral Outbreak of Chagas disease in Santa Catarina, Brazil: experimental evaluation of a patient’s strain. PLoS One (2015) 10:e0122566. 10.1371/journal.pone.0122566 26469517 PMC4607495

[71] Dias GB Gruendling AP Araujo SM Gomes ML Toledo MJ. Evolution of infection in mice inoculated by the oral route with different developmental forms of *Trypanosoma cruzi* I and II. Exp Parasitol (2013) 135:511–7. 10.1016/j.exppara.2013.08.013 23994765

[72] de Meis J Barreto de Albuquerque J Silva dos Santos D Farias-de-Oliveira DA Berbert LR Cotta-de-Almeida V *Trypanosoma cruzi* entrance through systemic or mucosal infection sites differentially modulates regional immune response following acute infection in mice. Front Immunol (2013) 4:216 10.3389/fimmu.2013.00216 23898334 PMC3724200

[73] Marsden PD *Trypanosoma cruzi* infections in CFI mice. II. Infections induced by different routes. Ann Trop Med Parasitol (1967) 61:62–7. 10.1080/00034983.1967.11686458 6051540

[74] Caradonna K Pereiraperrin M. Preferential brain homing following intranasal administration of *Trypanosoma cruzi*. Infect Immun (2009) 77:1349–56. 10.1128/IAI.01434-08 19168740 PMC2663175

[75] Eickhoff CS Dunn BA Sullivan NL Hoft DF. Comparison of the infectivity of *Trypanosoma cruzi* insect-derived metacyclic trypomastigotes after mucosal and cutaneous contaminative challenges. Mem Inst Oswaldo Cruz (2013) 108:508–11. 10.1590/S0074-02762013000400018 23828001 PMC3970623

[76] Ronco MT Frances DE Ingaramo PI Quiroga AD Alvarez ML Pisani GB Tumor necrosis factor alpha induced by *Trypanosoma cruzi* infection mediates inflammation and cell death in the liver of infected mice. Cytokine (2010) 49:64–72. 10.1016/j.cyto.2009.09.012 19892564

[77] Holscher C Mohrs M Dai WJ Kohler G Ryffel B Schaub GA Tumor necrosis factor alpha-mediated toxic shock in *Trypanosoma cruzi*-infected interleukin 10-deficient mice. Infect Immun (2000) 68:4075–83. 10.1128/IAI.68.7.4075-4083.2000 10858224 PMC101698

[78] Andrade SG Magalhaes Ldos A Pessina DH. Importance of TNF-alpha in the course of acute infection with *Trypanosoma cruzi*: influence of its inhibition by pentoxifylline treatment. Mem Inst Oswaldo Cruz (2008) 103:21–6. 10.1590/S0074-02762008005000006 18345460

[79] Rodrigues AA Notario AF Teixeira TL Silva E Quintal RT Alves AP A high throughput analysis of cytokines and chemokines expression during the course of *Trypanosoma cruzi* experimental oral infection. Acta Trop (2016) 157:42–53. 10.1016/j.actatropica.2016.01.025 26827742

[80] de Meis J Ferreira LM Guillermo LV Silva EM Dosreis GA Lopes MF. Apoptosis differentially regulates mesenteric and subcutaneous lymph node immune responses to *Trypanosoma cruzi*. Eur J Immunol (2008) 38:139–46. 10.1002/eji.200737582 18085669

[81] Reed SG Brownell CE Russo DM Silva JS Grabstein KH Morrissey PJ. IL-10 mediates susceptibility to *Trypanosoma cruzi* infection. J Immunol (1994) 153:3135–40. 8089491

[82] Blaschitz C Raffatellu M. Th17 cytokines and the gut mucosal barrier. J Clin Immunol (2010) 30:196–203. 10.1007/s10875-010-9368-7 20127275 PMC2842875

[83] Hu W Pasare C. Location, location, location: tissue-specific regulation of immune responses. J Leukoc Biol (2013) 94:409–21. 10.1189/jlb.0413207 23825388 PMC3747123

[84] D’Orazio SE. Animal models for oral transmission of *Listeria monocytogenes*. Front Cell Infect Microbiol (2014) 4:15. 10.3389/fcimb.2014.00015 24575393 PMC3920067

[85] Toso MA Vial UF Galanti N [Oral transmission of Chagas’ disease]. Rev Med Chil (2011) 139:258–66. 10.4067/S0034-98872011000200017 21773665

[86] Novak N Haberstok J Bieber T Allam JP. The immune privilege of the oral mucosa. Trends Mol Med (2008) 14:191–8. 10.1016/j.molmed.2008.03.001 18396104

[87] Weiner HL Da Cunha AP Quintana F Wu H. Oral tolerance. Immunol Rev (2011) 241:241–59. 10.1111/j.1600-065X.2011.01017.x 21488901 PMC3296283

[88] Moingeon P Mascarell L. Induction of tolerance via the sublingual route: mechanisms and applications. Clin Dev Immunol (2012) 2012:623474. 10.1155/2012/623474 22110534 PMC3216342

[89] Kursar M Bonhagen K Kohler A Kamradt T Kaufmann SH Mittrucker HW. Organ-specific CD4+ T cell response during *Listeria monocytogenes* infection. J Immunol (2002) 168:6382–7. 10.4049/jimmunol.168.12.6382 12055256

[90] Kursar M Kohler A Kaufmann SH Mittrucker HW. Depletion of CD4+ T cells during immunization with nonviable *Listeria monocytogenes* causes enhanced CD8+ T cell-mediated protection against listeriosis. J Immunol (2004) 172:3167–72. 10.4049/jimmunol.172.5.3167 14978123

[91] Sheridan BS Pham QM Lee YT Cauley LS Puddington L Lefrancois L. Oral infection drives a distinct population of intestinal resident memory CD8(+) T cells with enhanced protective function. Immunity (2014) 40:747–57. 10.1016/j.immuni.2014.03.007 24792910 PMC4045016

[92] Pepper M Linehan JL Pagan AJ Zell T Dileepan T Cleary PP Different routes of bacterial infection induce long-lived TH1 memory cells and short-lived TH17 cells. Nat Immunol (2010) 11:83–9. 10.1038/ni.1826 19935657 PMC2795784

